# Effect of Dual-Task Training on Cognitive Function in Community-Dwelling Older Adults With Mild Cognitive Impairment: Sequential Multiple Assignment Randomized Trial

**DOI:** 10.2196/79274

**Published:** 2025-11-25

**Authors:** Chenhao Yu, Dongsheng Bian, Jiatao Zhang, Xiao Han, Chenshu Shi, Guohong Li

**Affiliations:** 1 School of Public Health Shanghai Jiao Tong University School of Medicine Shanghai China; 2 School of Global Health Shanghai Jiao Tong University School of Medicine Shanghai China; 3 Department of Clinical Nutrition Ruijin Hospital Shanghai Jiao Tong University School of Medicine Shanghai China; 4 Institute of Healthy Yangtze River Delta Shanghai Jiao Tong University Shanghai China

**Keywords:** adaptive intervention, community prevention, dual-task intervention, dynamic treatment regimen, mild cognitive impairment

## Abstract

**Background:**

Nonpharmacological interventions are important prevention strategies for mild cognitive impairment (MCI), but effects vary significantly between individuals based on personal characteristics, while current practice relies on experience-based approaches lacking personalized, adaptive intervention strategies.

**Objective:**

The objective of our study was to develop and evaluate evidence-based adaptive intervention strategies for optimizing cognitive function among older adults with MCI using a Sequential Multiple Assignment Randomized Trial (SMART) design, comparing the effectiveness of cognitive training (CT) combined with virtual reality Taichi (VRTC) versus offline Taichi (OffTC) versus control, and to identify baseline characteristics that predict treatment response for personalized intervention delivery.

**Methods:**

We recruited 92 community-dwelling adults aged ≥60 years diagnosed with MCI from 3 districts in Shanghai, China. A 24-week SMART was conducted between April and December 2023. During the first stage (weeks 1-12), participants were randomly assigned to control (n=26) or intervention groups receiving CT combined with either OffTC (n=33) or VRTC (n=34). Nonresponders at week 12 were rerandomized to alternative or intensified interventions during the second stage (weeks 13-24). The primary outcome was the Memory Guard score (MGs) at 24 weeks. Dynamic treatment regimen analysis assessed optimal adaptive strategies using regression models.

**Results:**

A total of 81 participants completed the trial. CT+VRTC demonstrated significantly superior cognitive improvement compared to control (5.10 MGs, 95% CI 2.93-7.27; Cohen *d*=1.425, 95% CI 0.785-2.060; *P*<.001) and CT+OffTC (3.61 MGs, 95% CI 1.71-5.51; Cohen *d*=1.009, 95% CI 0.461-1.560; *P*<.001). At 24 weeks, adjusted mean MGs were: CT+VRTC 32.9 (95% CI 31.3-34.5), CT+OffTC 29.3 (95% CI 27.7-30.9), and control 27.8 (95% CI 26.0-29.6). Dynamic treatment regimen analysis revealed VRTC-based adaptive strategies consistently outperformed static approaches, with VRTC responders achieving the highest effectiveness (5.40 MGs improvement, 95% CI 3.10-7.70; *P*<.001). Treatment intensification proved more effective than modality switching for nonresponders. Subgroup analyses revealed that younger participants (≤71 years), individuals with lower baseline cognitive function, and those with comorbid conditions demonstrated enhanced responsiveness, suggesting these populations may derive greater benefit from virtual reality–based approaches.

**Conclusions:**

This SMART trial established the first evidence-based adaptive intervention framework for community MCI prevention, demonstrating that virtual reality–enhanced Taichi combined with CT produced superior outcomes compared with traditional exercise and control conditions. Treatment intensification proved more effective than modality switching for nonresponders. Diabetes showed a better response enabling personalized intervention selection. These findings provide clinicians with objective decision rules for treatment adaptation and identify high-benefit populations (younger adults, those with lower baseline cognition, and patients with metabolic comorbidities) for targeted intervention. The protocol can be implemented by community health workers within existing urban health care infrastructure, offering a scalable approach to precision-based cognitive health management in aging populations.

**Trial Registration:**

Chinese Clinical Trial Registry ChiCTR2100042748; https://www.chictr.org.cn/showproj.html?proj=120941

## Introduction

The increased life expectancy is one of the greatest achievements of global health systems. However, this increase has led to a substantial rise in age-related neurological disorders, particularly Alzheimer disease and other dementias, necessitating health policies to focus [1], not only on survival but also on minimizing health loss due to disability by promoting function and independence [2]. Estimation suggested an increase from 57 million people living with dementia in 2019 to 153 million by 2050 [3]. Between 1990 and 2021, the disability-adjusted life years attributed to dementia increased by 168.7% (95% CI 156.3-179.9), driven by population aging [4]. By 2021, dementia ranked as one of the top 10 neurological conditions globally, with a particularly high burden in people aged 80 years and older [5]. Mild cognitive impairment (MCI) is a bidirectional transitional stage between dementia and normal cognitive aging, often with unrecognized early symptoms, with 10%-15% of affected individuals progressing to dementia annually [6]—a risk 3.3 times higher than that of the general older population [7]. Therefore, MCI represents a critical window for dementia prevention and intervention, with early identification and intervention being essential for reducing dementia incidence. Achieving a cost-effective community intervention has become a critical priority in public health [3].

Exercise intervention and cognitive training (CT) can effectively improve cognitive functions [8]. Mind-body exercise, such as Taichi and yoga, is a promising exercise type incorporating physical, cognitive, social, and mindfulness components in exercise [9] that may have a better cognitive management effect than other types of exercise [10].

Recent evidence suggests that both mind-body exercises and technology-enhanced interventions show promise for MCI prevention [10]. Systematic reviews demonstrate that Taichi significantly improves cognitive function in older adults with MCI, particularly executive function and memory [11]. Similarly, virtual reality (VR) technology has emerged as a powerful cognitive rehabilitation tool, offering ecological validity and real-time adaptability that traditional training lacks. VR-based interventions demonstrate significant improvements in memory, attention, and executive function in populations with MCI [12,13]. However, fewer studies synerized the potential effects of combining traditional mind-body exercise with VR technology using adaptive intervention strategies.

Systematic reviews and meta-analyses have shown that dual-task intervention combining cognitive function training and physical activity is more effective in improving cognition and physical function than physical activity intervention [14,15]. This synergistic effect is explained by the guided plasticity facilitation framework, whereby exercise facilitates neuroplasticity by increasing brain-derived neurotrophic factor production and cell proliferation, while cognitive interventions guide this plasticity by enhancing the survival of exercise-induced new cells. Additionally, multicomponent exercise research confirms positive effects on global cognition (effect size=0.32, 95% CI 0.03-0.61), particularly when aerobic exercise is included, supporting the mechanistic basis of the muscle-brain axis in exercise-induced neuroprotection [16,17].

The Healthy Ageing Through Internet Counselling in the Elderly (HATICE) trial is a multinational preventive intervention study based on coaching support and is a low-cost and scalable intervention model. HATICE provides remote personalized adaptive intervention through coaching, reduces the burden of cardiovascular disease, and improves cognitive function [18]. STRONGER 60+ also pays attention to the need for adaptive intervention. This model is based on the multicomponent intervention of The Finnish Geriatric Intervention Study to Prevent Cognitive Impairment and Disability (FINGER) [19,20], but focuses more on how to achieve adaptive intervention [21]. However, current clinical guidelines and expert consensus on multidomain intervention for MCI and dementia prevention provide a range of content and duration that is too wide to make the specific guidance for adaptation in community and clinical practice [1,7,22,23], and the current training components and duration of multidomain intervention still depend on experience and experience-based approaches [24].

The dose-response relationship study of CT found that there is an optimal point for the intervention duration, and the optimal duration of the intervention is affected by the age of the participants [25]. Observational studies have also found that age and comorbidities affect the prevention and progression of MCI [26,27]. Meanwhile, inappropriate strategy selection might reduce treatment adherence and compromise the critical intervention window [28]. Therefore, how to achieve adaptive intervention, explore which factors, and formulate adaptive intervention duration (or doses) based on these factors are the key to nonpharmacological prevention of MCI and remain to be developed.

We used a 2-stage sequential, multiple assignment, randomized trial (SMART) design to develop evidence-based adaptive intervention strategies [29] for optimizing cognitive function among older adults with MCI. The SMART design involved CT combined with either offline Taichi (OffTC) or virtual reality Taichi (VRTC). Participants who did not respond adequately to initial treatment (early treatment nonresponders) were rerandomized to receive either treatment intensification or modified treatment components.

The primary aim was to compare the effectiveness of CT+OffTC versus CT+VRTC versus control on long-term cognitive outcomes. Among early treatment nonresponders, we evaluated whether treatment intensification or component modification produced superior cognitive improvements. We hypothesized that both active interventions would demonstrate greater cognitive benefits compared with control and that treatment intensification would be more effective than component modification among nonresponders. The secondary aims assessed baseline characteristics that predict treatment response and effect modification. We hypothesized that age, baseline cognitive function, and comorbid conditions would influence both response status and treatment effectiveness.

## Methods

### Study Design and Setting

The study was conducted in 3 randomly selected districts in Shanghai between April and December 2023. A multistage stratified cluster random sampling method was used for participant recruitment. Based on geographical location and economic development level, 3 districts were randomly selected from the Shanghai municipality. Within each selected district, 2 subdistricts were randomly chosen, and subsequently, 3 communities were randomly selected from each subdistrict using cluster sampling. This sampling approach ensured geographical and socioeconomic diversity in the study population while maintaining feasibility for intervention delivery through existing community health service infrastructure.

This study was reported according to the Consolidated Standards of Reporting Trials (CONSORT 2025) for randomized trials, detailed in Multimedia Appendix 1 [30].

### Treatments

OffTC intervention implemented the standardized 24-form simplified Taichi exercise regimen, developed by the National Sports Administration. Modifications were implemented for balance-intensive movements (right kick, double ear boxing, left turning kick, left standing stance, and right standing stance) to enhance safety while maintaining form integrity.

The VRTC intervention used identical 24-form simplified Taichi movements implemented through an immersive technological interface. The VR system incorporated precise arm-length calibration to accurately track upper and lower extremity movement trajectories.

CT used the Thoven Cognitive Training System (TCSA-BOT, developed by Shanghai Thoven Intelligent Technology Co, Ltd), a comprehensive platform targeting multiple cognitive domains: memory, attention, executive function, logical reasoning, and reaction time. The system was specifically adapted for this research protocol and implemented via a customized WeChat mini-program interface.

### Procedures

This study used a 24-week SMART design, comprising two 12-week intervention phases. In the first stage, the intervention consisted of 1 hour of weekly CT combined with 1 hour of weekly exercise per week, structured as a dual-task training program incorporating both cognitive and physical elements.

Participants were initially randomized into 3 groups: control group, CT+OffTC, and CT+VRTC. At week 12, participants were assessed based on 2 questions assessed by a 5-point Likert scale: (1) perceived effectiveness of their current intervention and (2) proficiency in both CT and Taichi exercises. Participants would be marked as nonresponders, if any question scored less than 3 points.

For the second phase, CT+OffTC responders continued their original intervention protocol. Nonresponders from CT+OffTC were randomly assigned to either CT+VRTC or doubled doses of their original intervention (2CT+2OffTC). Similarly, CT+VRTC responders maintained their initial intervention, while nonresponders were randomly assigned to either CT+OffTC or doubled doses of their original intervention (2CT+2VRTC).

All interventions were conducted at designated community health centers within the 3 participating districts in Shanghai. OffTC sessions were led by certified Yang-style Taichi instructors with a minimum of 5 years of experience teaching older adults, while VRTC sessions used Meta Quest with Guided Tai Chi software (developer: Cubicle Ninjas) providing immersive virtual environments featuring professionally recorded Yang-style Taichi instruction and real-time movement tracking without requiring external controllers or television displays. Each VRTC session included 5-minute acclimatization periods and integrated safety features. CT was delivered through the TCSA-BOT WeChat mini-program interface, with research staff supervision during weekly 60-minute community center sessions and optional home access via participants’ smartphones. Control group health education consisted of weekly 45-minute group sessions covering general wellness topics, delivered by qualified health educators in the same community facilities, detailed intervention program in Multimedia Appendix 2 and VRTC sample video in Multimedia Appendix 3.

### Randomization and Masking

After baseline assessment, participants were randomly assigned to the intervention groups or the control groups. In the first 12 weeks of the 24-week SMART intervention, participants in the intervention group were randomly assigned to receive either weekly 1-hour CT plus 1-hour Taichi or CT plus VRTC. In the 12th week, participants were assessed for intervention mastery, with nonresponders those who are unable to master the content of the intervention, the Likert 5-point scale evaluates the mastery of the intervention content to be less than 3, being rerandomized to alternative or intensified interventions. The control group received only health education and routine care. Randomization was performed with the use of a computer-based code generated by members of the research team at the School of Public Health at the Shanghai Jiao Tong University School of Medicine. Due to the dosage assignment of the intervention being changed during treatment, study participants and staff were aware of treatment allocation, but researchers responsible for data analysis were masked to the allocation groups.

### Participants

Participants were recruited from 3 randomly selected districts in Shanghai. We screened community-dwelling older adults using the Montreal Cognitive Assessment (MoCA) and Memory Guard score (MGs).

Study eligibility criteria included: age ≥60 years, MCI diagnosis established through positive screening on the MGs, and meeting the 2018 Chinese guidelines for MCI diagnosis [23]. Exclusion criteria encompassed: communication disorders, severe impairment in activities of daily living, presence of metal implants, severe psychiatric disorders, illiteracy, cognitive decline attributable to other pathologies, history of neurological diseases, exercise contraindications, color blindness, insufficient education to complete testing protocols, concurrent rehabilitation therapy, or unwillingness to complete the 24-week intervention and follow-up. Additionally, participants with regular exercise experience (eg, Taichi and Baduanjin) within the preceding 3 months were excluded to prevent contamination effects.

### Assessments and Data Sources

#### Outcome Measurements

The primary outcome is cognitive status at 24 weeks (end of the trial), measured by MGs. MoCA was used for baseline cognitive screening and participant categorization.

The MoCA is a widely validated cognitive screening instrument that assesses multiple cognitive domains including visuospatial and executive functions, naming, memory, attention, language, abstraction, delayed recall, and orientation [31]. The MoCA uses a 30-point scale, with scores ≤26 indicating possible cognitive impairment [29]. In our study [23], MoCA was administered at baseline to categorize participants into cognitive risk groups.

MGs is a computerized neuropsychological assessment device that evaluates 6 cognitive domains: orientation, memory, attention, calculation, recall, and language and executive function [32]. The assessment incorporates both accuracy scores (correct or incorrect responses) and response time data, using machine learning algorithms to provide a comprehensive cognitive evaluation. MGs demonstrates excellent diagnostic performance with an accuracy of 93.75% and an area under the curve of 0.923, achieving high sensitivity (91.67%) and specificity (95.45%) for MCI detection [32]. The interassessment agreement between MoCA and MGs reached κ=1.0, indicating perfect concordance and validating the reliability of our cognitive measurements. The computerized format allows for standardized administration, objective measurement of cognitive performance, and real-time data collection, making it particularly suitable for detecting cognitive changes in intervention studies.

#### Exposures

The primary exposures in this study were the intervention modalities assigned through the 2-stage SMART design: control, CT+OffTC, and CT+VRTC. Detailed descriptions of each intervention component, delivery methods, and the rerandomization process for nonresponders have been provided in the “Procedures” section above.

#### Confounders

Potential confounding variables were identified based on existing literature on cognitive function interventions and measured at baseline through standardized protocols by trained research staff. Demographic characteristics included age (continuous variable in years, assessed through participant self-report), sex (binary: male or female, obtained from participant demographics), and education level (continuous variable in years). Socioeconomic status was measured through self-reported monthly household income, categorized in Chinese yuan (¥; US $1= ¥7.11).

Clinical characteristics assessed at baseline included BMI (in kg/m², calculated from measured height and weight using calibrated equipment). Comorbid conditions were assessed as binary variables (yes or no) through self-report, including diabetes (assessed through physician diagnosis or current use of antidiabetic medications), hypertension (assessed through physician diagnosis or current use of antihypertensive medications), cancer, cardiovascular disease, chronic kidney disease, fracture history, respiratory disease, and digestive disease.

### Sample Size

The intervention was designed to find the optimal adaptive intervention based on individual responses to initial treatment assignments. The sample size of the SMART design relied on the response rate. Response rate indicates whether the intervention is effective. According to the previous studies on physical activity intervention [15,33] and response rate simulation [34,35], we hypothesized the response rate of 0.60, the type I error rate of 0.05, a desired half-width of the CI of 0.45, and the required sample size of SMART intervention group is 54 [36] based on precision-based sample size calculation.

### Statistical Analysis

Baseline characteristics were summarized using descriptive statistics, with continuous variables presented as means (SD) and categorical variables as frequencies (percentages). Group comparisons were performed using *t* test for normally distributed continuous variables and the chi-square test for categorical variables.

The primary analysis used intention-to-treat principles comparing 24-week memory scores across treatment groups (CT+OffTC, CT+VRTC, and control) using linear regression adjusted for baseline MGs, age, education, sex, hypertension, and diabetes. Pairwise comparisons between groups were conducted with Bonferroni correction for multiple testing. Effect sizes were calculated as Cohen *d* with 95% CIs.

Secondary analyses evaluated treatment strategies among early treatment nonresponders (defined as insufficient memory improvement at 12 weeks) using linear regression models comparing treatment intensification versus switching Tai Chi modality, adjusted for 12-week memory scores and baseline characteristics. Dynamic treatment regimen analysis compared embedded treatment sequences using the same covariate-adjusted linear regression approach. Treatment response prediction used linear regression and logistic regression models to identify baseline characteristics associated with 12-week response status. Subgroup analysis was assessed using interaction terms for age group, baseline memory function, hypertension, and diabetes status.

All statistical tests were 2-sided with a significance threshold of *P*<.05. Bootstrap resampling (1000 iterations) was used to construct 95% CIs and assess statistical significance.

### Ethical Considerations

This study was reviewed and approved by the Public Health and Nursing Research Ethics Committee of Shanghai Jiao Tong University School of Medicine (approval number: SJUPN-202008, on November 19, 2020) prior to participant recruitment and registered with the Chinese Clinical Trial Registry on January 27, 2021, ChiCTR2100042748. The study protocol, informed consent procedures, and all study materials received full ethical approval.

Written informed consent was obtained from all participants prior to enrollment. The consent process included detailed explanations of (1) study purpose, procedures, and duration; (1) potential risks and benefits of participation; (3) voluntary nature of participation and right to withdraw at any time without penalty; (4) data collection, storage, and use procedures; and (5) contact information for study personnel and ethics committee. All participants demonstrated the capacity to provide informed consent through cognitive screening assessments.

All study data were deidentified using unique participant identification codes, with the linking key stored separately from study data in a secure, password-protected database accessible only to authorized research personnel. Data analysis was conducted using only deidentified datasets.

Participants received modest compensation for their time and effort, including transportation reimbursement (¥50 [US $7] per visit; currency conversion based on exchange rate as of October 8, 2025) for assessment sessions and a completion incentive (¥200 [US $28] for those who finished the full 24-week intervention period. Compensation was provided regardless of intervention adherence or study outcomes to avoid coercion.

No images containing identifiable participant information are included in this manuscript or supplementary materials.

Harms including falls, heat exhaustion, dizziness, and VR-related symptoms were assessed nonsystematically through participant self-report and instructor observation throughout the 24-week intervention period.

## Results

### Descriptive Statistics

Between April and December 2023, a total of 686 individuals were assessed for eligibility, of whom 563 were screened as ineligible due to normal cognitive function. Of the 123 participants who met eligibility criteria, 30 declined participation due to COVID-19 pandemic concerns regarding in-person study visits and health safety protocols, and 1 withdrew prior to randomization, resulting in 92 participants entering Stage I randomization. Participants were allocated to 3 groups: 26 to control, 33 to OffTC+CT, and 33 to VRTC+CT.

**Figure 1:**
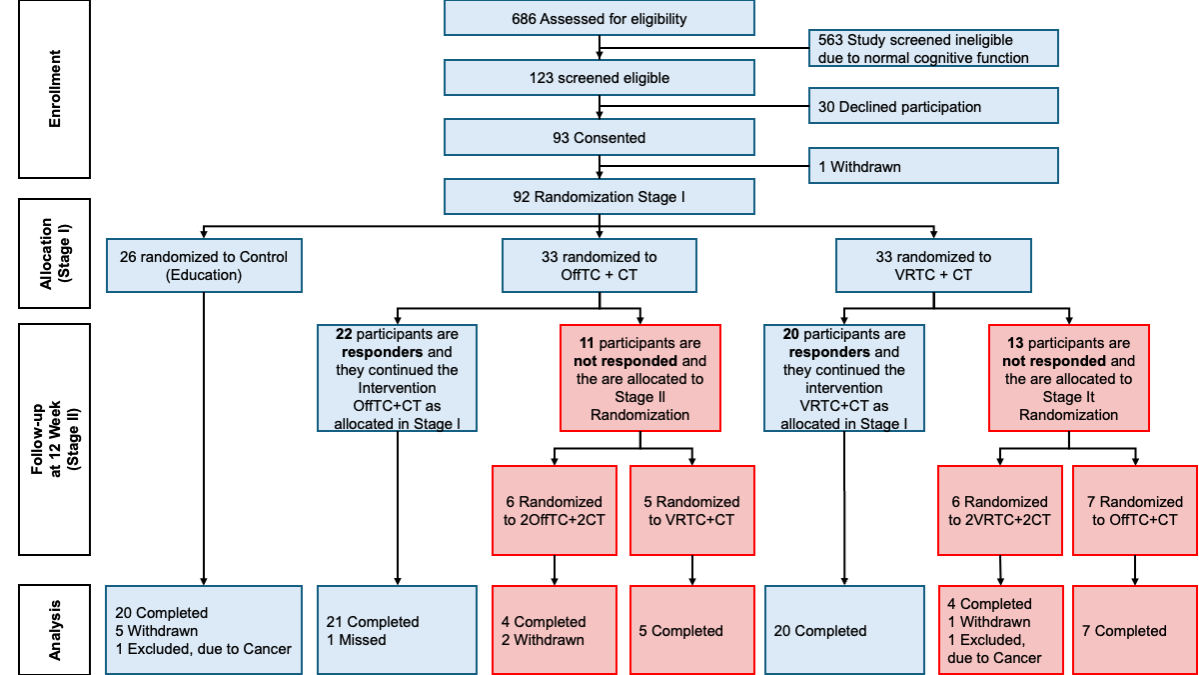
Consolidated Standards of Reporting Trials (CONSORT) flow diagram of participant recruitment, randomization, and retention in a 24-week Sequential Multiple Assignment Randomized Trial examining cognitive training combined with offline Taichi versus virtual reality Taichi for mild cognitive impairment prevention among community-dwelling adults aged ≥60 years in Shanghai, China (April-December 2023).

Following Stage I intervention, responders continued their allocated treatments while nonresponders proceeded to Stage II randomization with alternative interventions. In the control group, 20 participants completed the study with 6 withdrawals. Among OffTC+CT participants, 22 responders continued treatment with 21 completing it, while 11 nonresponders were randomized to alternative treatments (6 to 2OffTC+2CT and 5 to VRTC+CT). In the VRTC+CT group, 20 responders completed treatment, while 13 nonresponders underwent Stage II randomization (6 to 2VRTC+2CT and 7 to OffTC+CT) with completion rates of 4/6 and 7/7, respectively (Figure 1).

The final sample consisted of 81 participants, and the response rate was 64.18% which met the assumption of sample calculation. The study population was predominantly female (65.4% overall), with similar gender distributions across groups (60.0%, 66.7%, and 67.7%, respectively; *P*=.84), detailed in Table 1. All interventions were delivered as intended by certified instructors showing >95% fidelity to standardized procedures.

**Table 1:** Baseline demographic, socioeconomic, and clinical characteristics of community-dwelling adults aged ≥60 years with mild cognitive impairment enrolled in a 24-week Sequential^a^.

Variables		Overall	Control		OffTC^b^+CT^c^		VRTC^d^+CT		*P* value	
Observation		81	20		30		31			
Female sex, n (%)		53 (65.4)	12 (60.0)		20 (66.7)		21 (67.7)		.83	
Education (years), mean (SD)		10.55 (2.94)	11.10 (3.60)		10.63 (3.02)		10.11 (2.39)		.50	
**Income, CNY (USD), n (%)**										.53
	< 10,000 (< 1430)	1 (1.2)		0 (0.0)		1 (3.3)		0 (0.0)		
	10,001-30,000 (1431-4285)	12 (14.8)	5 (25.0)		5 (16.7)		2 (6.5)			
	30,001-60,000 (4286-8570)	49 (60.5)	11 (55.0)		18 (60.0)		20 (64.5)			
	60,001-90,000 (8571-12,855)	11 (13.6)	3 (15.0)		4 (13.3)		4 (12.9)			
	90,001 120,000 (12,856-17,140)	6 (7.4)	0 (0.0)		2 (6.7)		4 (12.9)			
	> 120,001 (> 17,141)	2 (2.5)	1 (5.0)		0 (0.0)		1 (3.2)			
Age (years), mean (SD)		71.38 (6.49)	72.75 (7.53)		72.37 (6.88)		69.55 (5.00)		.13	
Diabetes, n (%)		11 (13.6)	4 (20.0)		4 (13.3)		3 (9.7)		.57	
Hypertension, n (%)		24 (29.6)	8 (40.0)		8 (26.7)		8 (25.8)		.50	
BMI, mean (SD)		24.06 (2.75)	24.87 (2.58)		23.75 (3.03)		23.83 (2.56)		.31	
MGs^e^, mean (SD)		27.61 (3.55)	27.99 (3.31)		27.25 (4.31)		27.72 (2.92)		.75	
MoCA^f^, mean (SD)		22.19 (2.15)	21.50 (2.28)		22.20 (2.22)		22.61 (1.93)		.19	
Cancer, n (%)		8 (9.9)	2 (10.0)		1 (3.3)		5 (16.1)		.24	
Cardiovascular disease, n (%)		9 (11.1)	1 (5.0)		3 (10.0)		5 (16.1)		.45	
Chronic kidney disease, n (%)		2 (2.5)	0 (0.0)		1 (3.3)		1 (3.2)		.71	
Fracture, n (%)		8 (9.9)	3 (15.0)		2 (6.7)		3 (9.7)		.62	
Respiratory disease, n (%)		3 (3.7)	0 (0.0)		3 (10.0)		0 (0.0)		.07	
Digestive disease, n (%)		3 (3.7)	0 (0.0)		1 (3.3)		2 (6.5)		.48	

^a^Groupwise comparisons for continuous variables were assessed using either ANOVA or the Kruskal-Wallis test (if not normally distributed), and categorical variables were assessed using the chi-square test.

^b^OffTC: offline Taichi.

^c^CT: cognitive training.

^d^VRTC: virtual reality Taichi.

^e^MGs: Memory Guard score.

^f^MoCA: Montreal Cognitive Assessment.

### Effect of Interventions

At 12 weeks, the adjusted mean MGs in the CT+VRTC group were 30.1 (95% CI 28.3-31.9) and 28.1 (95% CI 26.3-29.9) in the CT+OffTC group. The effect of the CT+VRTC group was significantly better than that of the CT+OffTC group, with an increase of 2.03 MGs (95% CI 0.018-3.96; Cohen *d*=0.558; *P*=.04; Tables 2 and 3).

**Table 2:** Treatment effects on Memory Guard scores at 12 weeks and 24 weeks in a Sequential Multiple Assignment Randomized Trial of cognitive training combined with offline Taichi (CT+OffTC) versus virtual reality Taichi (CT+VRTC) versus control among community-dwelling adults aged ≥60 years with mild cognitive impairment^a^.

Timepoint and treatment group		Adjusted mean (SE)	95% CI	Group comparison	Estimate (SE)	t-ratio	*P* Value	Effect size (95% CI)
**Week 12**								
	CT+OffTC	28.1 (0.907)	26.3- 29.9	CT+OffTC vs CT+VRTC	–2.03 (0.961)	–2.117	.04	–0.558 (–1.1 to –0.018)
	CT+VRTC	30.1 (0.900)	28.3-31.9	—^b^	—	—	—	—
**Week 24**								
	Control	27.8 (0.903)	26.0 - 29.6	CT+OffTC vs Control	1.49 (1.050)	1.421	.47	0.416 (–0.171 to 1.00)
	CT+OffTC	29.3 (0.803)	27.7 -30.9	CT+VRTC vs Control	5.10 (1.070)	4.778	<.001	1.425 (0.785- 2.06)
	CT+VRTC	32.9 (0.806)	31.3 -34.5	CT+VRTC vs CT+OffTC	3.61 (0.936)	3.857	<.001	1.009 (0.461- 1.56)

^a^Adjusted mean Memory Guard scores, pairwise group comparisons, and effect sizes (Cohen *d*) are presented from linear regression models controlling for baseline Memory Guard scores, age, education, sex, hypertension, and diabetes.

**Table 3:** Comparison of treatment effects on Memory Guard scores at 12 weeks and 24 weeks in a Sequential Multiple Assignment Randomized Trial of cognitive training combined with offline Taichi (CT+OffTC) versus virtual reality Taichi (CT+VRTC) versus control among community-dwelling adults aged ≥60 years with mild cognitive impairment.^a^

Time point and group comparison			Estimate (SE)		t-ratio		*P* Value		Effect size (95% CI)
**Week 12**									
	CT+OffTC vs CT+VRTC	–2.03 (0.961)		–2.117		.04		–0.558 (–1.1 to –0.018)	
**Week 24**									
	CT+OffTC vs Control	1.49 (1.050)		1.421		.47		0.416 (–0.171 to 1.00)	
	CT+VRTC vs Control	5.10 (1.070)		4.778		<.001		1.425 (0.785- 2.06)	
	CT+VRTC vs CT+OffTC	3.61 (0.936)		3.857		<.001		1.009 (0.461- 1.56)	

^a^Adjusted mean Memory Guard scores, pairwise group comparisons, and effect sizes (Cohen *d*) are presented from linear regression models controlling for baseline Memory Guard scores, age, education, sex, hypertension, and diabetes.

At 24 weeks, the adjusted mean MGs in the CT+VRTC group was the highest, at 32.9 (95% CI 31.3-34.5), followed by the CT+OffTC group, at 29.3 (95% CI 27.7-30.9), and the control group at 27.8 (95% CI 26.0-29.6).

In pairwise comparisons, the CT+VRTC group showed an improvement of 5.10 (95% CI 2.93-7.27; Cohen *d*=1.425, 95% CI 0.785-2.060; *P*<.001) MGs compared with the control group, while the CT+VRTC group showed an improvement of 3.61 (95% CI 1.71-5.51; Cohen *d*=1.009, 95% CI 0.461-1.560; *P*<.001) MGs compared with the CT+OffTC group.

The embedded dynamic treatment plan analysis showed that the efficacy of different adaptive strategies was different (Table 4). The VRTC-based plan was always more effective than the control group, among which the responder plan had the highest efficacy, with an efficacy difference of 5.40 (95% CI 3.10-7.70; *P*<.001) MGs compared with the control group. VRTC intensification showed a significant benefit for nonresponders, with a difference of 5.66 (95% CI 1.14-10.18; *P*=.01) MGs, while VRTC switch showed a difference of 3.21 (95% CI 0.01-6.41; *P*=.05) MGs. Among the OffTC-based strategies, only OffTC intensification showed significant improvement compared with the control group, with a difference of 4.24 (95% CI 0.22-8.26; *P*=.04) MGs.

**Table 4:** The comparisons of dynamic treatment regimen strategies on the 24-week Memory Guard score by treatment response and intervention sequence^a^.

Strategies	Adjusted mean (SE)	95% CI	Comparisons	*P* value
Control	27.6 (1.35)	24.9-30.3	Reference	—^b^
OffTC^c^ Intensify	31.8 (1.89)	28.1-35.6	4.24 (2.01)	.04
OffTC Switch	29.0 (1.70)	25.6-32.4	1.37 (1.83)	.45
OffTC Responder	28.4 (1.13)	26.2-30.7	0.84 (1.14)	.46
VRTC^d^ Intensify	33.3 (2.13)	29.0-37.5	5.66 (2.26)	.01
VRTC Switch	30.8 (1.54)	27.7-33.9	3.21 (1.60)	.05
VRTC Responder	33.0 (1.13)	30.7-35.2	5.40 (1.15)	<.001

^a^Adjusted means are from models controlling for baseline Memory Guard score, age, and other control variables.

^b^Not applicable.

^c^OffTC: offline Taichi.

^d^VRTC: virtual reality Taichi.

The results suggest that VRTC-based adaptive interventions provide strong cognitive benefits across response patterns, while OffTC-based approaches show more limited effectiveness, with benefits primarily seen when treatment intensity is increased in nonresponders.

### Factors Predicting the Response Status

Table 5 presents the results of response status predictors. For the response status, diabetes was a strong positive predictor (B=17.404, 95% CI 15.913-18.895; *P*<.001). Baseline MGs, treatment assignment in stage 1, hypertension, and age were not significant. For perceived effectiveness and content mastery, diabetes remained a significant positive predictor as the same. Additionally, lower baseline MGs was associated with higher perceived effectiveness and content mastery.

**Table 5:** Association between baseline characteristics and treatment response outcomes at 12 weeks among participants receiving cognitive training combined with Taichi interventions^a^.

						(1) Response			(2) Perceived effectiveness		(3) Master content		
**Baseline MGs^b^**													
		Unstandardized regression coefficient (95% CI)				–0.146 (–0.365 to 0.073)			–0.124^c^ (–0.245 to –0.004)		–0.115^c^ (–0.225 to –0.004)		
		Robust SE				–0.604			–0.202		–0.151		
	*P* value				.19			.05				.05	
**Treatment in stage 1**													
		Unstandardized regression coefficient (95% CI)				–0.061 (–1.331 to 1.208)			–0.009 (–0.818 to 0.800)		0.122 (–0.675 to 0.918)		
		Robust SE				–2.043			–0.599		–0.362		
	*P* value		.93					.98		.77			
**Age**													
		Unstandardized regression coefficient (95% CI)				0.001 (–0.120 to 0.122)			–0.023 (–0.105 to 0.058)		0.005 (–0.067 to 0.077)		
		Robust SE				–0.373			–0.163		–0.123		
	*P* value		.99					.57		.90			
**Hypertension**													
		Unstandardized regression coefficient (95% CI)				–1.209^d^ (–2.606 to 0.188)	–0.939^d^ (–1.903 to 0.025)				–0.768 (–1.783 to 0.248)		
		Robust SE				–0.160	–0.044				–0.109		
		*P* value		0.09					.06				.15
**Diabetes**													
		Unstandardized regression coefficient (95% CI)				17.404^e^ (15.913 to 18.895)			1.788^e^ (0.927 to 2.649)		1.598^e^ (0.705 to 2.492)		
		Robust SE				0.360			0.335		0.284		
		*P* value		<.001					<.001				.001
**Constant**													
		Unstandardized regression coefficient (95% CI)				4.926 (–8.173 to 18.026)			8.968^c^ (0.793 to 17.143)		6.561^d^ (–0.550 to 13.672)		
		Robust SE				44.669			17.395		13.164		
		*P* value		.46					.04		.08		
Observations, n						61			61		61		
*R* ^2^						—^f^			0.168		0.165		

^a^Values represent unstandardized regression coefficients with 95% CIs and robust SEs in parentheses. Model (1): Logistic regression; Models (2-3): Ordinary Least Squares regression. Robust SEs in parentheses.

^b^MGs: Memory Guard score.

^c^*P*<.05.

^d^*P*<.10.

^e^*P*<.01.

^f^Not applicable.

### Subgroup Analysis

**Figure 2:**
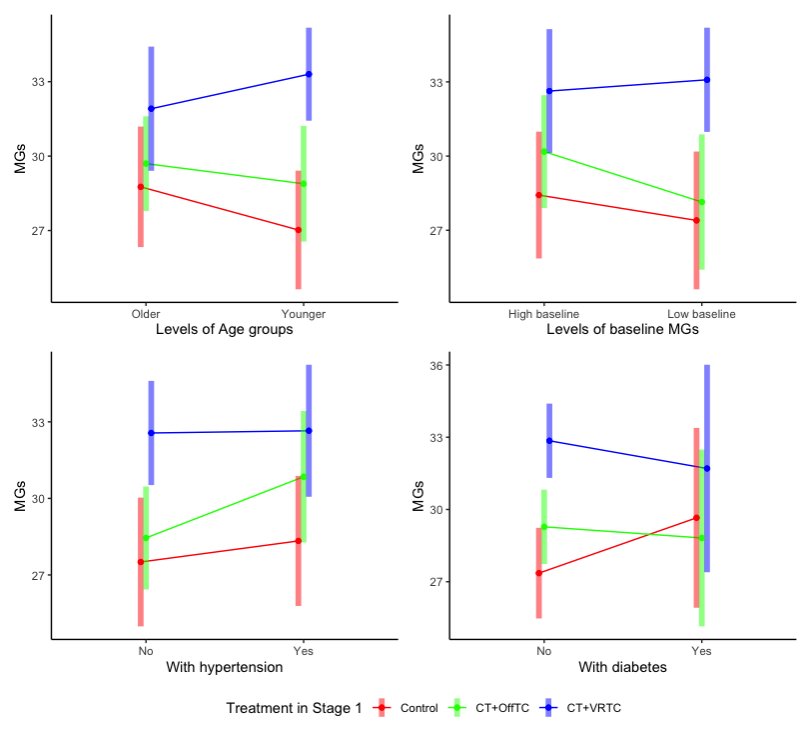
Subgroup analyses examining treatment effect heterogeneity on 24-week Memory Guard scores by age group (≤71 vs >71 years), baseline cognitive function (lower vs higher Memory Guard score), and comorbidity status (hypertension and diabetes) in a Sequential Multiple Assignment Randomized Trial comparing control, cognitive training combined with offline Taichi (CT+OffTC), and cognitive training combined with virtual reality Taichi (CT+VRTC) . Adjusted means with 95% CIs from linear regression models controlling for baseline Memory Guard score, age, and other covariates. MGs: Memory Guard score.

Subgroup analyses revealed significant heterogeneity in treatment effects (Figure 2), with younger age, lower baseline cognitive function, and the presence of comorbidities benefiting more from interventions.

Younger participants (≤71 years) benefit more cognitive improvement from CT+VRTC, achieving 33.3 (95% CI 31.4-35.2) compared with older participants at 31.9 (95% CI 29.4-34.4). Yet, younger patients with MCI were at greater risk of cognitive decline. This result suggested that younger patients with MCI may be more vulnerable to cognitive decline but also benefit more from VR interventions.

CT+VRTC demonstrated consistent effectiveness across different baseline cognitive levels. However, participants with higher baseline MGs showed greater overall benefit from interventions.

CT+VRTC maintained stable effectiveness regardless of comorbid conditions. Importantly, participants with comorbid conditions would benefit more cognitive improvement compared with those without comorbidities, suggesting potential synergy between controlling chronic diseases and cognitive function protection.

## Discussion

### Principal Results

CT+VRTC demonstrated substantial cognitive improvement compared with both control and CT+OffTC groups, with large effect sizes indicating clinically meaningful benefits. Dynamic treatment regimen analysis revealed that VRTC-based adaptive strategies consistently outperformed control across different response patterns, with VRTC responders achieving the highest effectiveness. Subgroup analyses identified significant treatment heterogeneity, with younger participants, those with lower baseline cognitive function, and individuals with comorbid conditions demonstrating enhanced responsiveness to interventions. To our knowledge, this is the first SMART design to develop evidence-based adaptive intervention strategies for MCI prevention in community settings.

### Comparison With Prior Work

Compared with the FINGER trial [19,20], our focused population with MCI demonstrated substantially greater cognitive improvements, with CT+VRTC achieving an effect size of 1.425 (95% CI 0.785-2.060; *P*<.001) compared with control, suggesting that technology-enhanced dual-task training may be effective in individuals with established cognitive impairment. Consistent with emerging digital health interventions, our findings support the superiority of VR-enhanced approaches over traditional exercise modalities [37-39], with VRTC consistently outperforming OffTC across all treatment strategies. The effects of VRTC suggest unique advantages of VR technology in cognitive intervention.

The immersive, multisensory environment inherent in VR-based training appears to impose greater demands on sensorimotor integration and movement precision, potentially enhancing cognitive engagement through increased activation of frontoparietal networks [40]. Furthermore, the enhanced real-time feedback mechanisms in VR environments may facilitate neuroplastic adaptations, particularly in frontocortical regions critical for executive function and motor planning [41]. In addition, VRTC may strengthen cortical connectivity and increase frontal lobe activation patterns, via upregulation of brain-derived neurotrophic factor in hippocampal and prefrontal regions, enhanced recruitment of dorsal and ventral attentional networks, increased cerebellum-prefrontal functional connectivity, and modulation of default mode network activity to reduce age-related network dedifferentiation [12,42,43]. Individuals who participated in the technology-enhanced dual-task group (CT+VRTC) showed more stable intervention effects regardless of whether they had chronic diseases or not and their baseline cognitive status.

The enhanced treatment responsiveness observed in participants with comorbidities reflects potential synergistic effects between intervention modalities and chronic disease management. Patients with comorbidities benefited more, which is consistent with the findings of current intervention studies [44,45]. Hypertension can lead to white matter injury through small vessel disease in the brain, while diabetes is associated with insulin resistance, inflammatory responses, and oxidative stress, all of which contribute to neurodegenerative processes [45]. Therefore, cognitive impairment in these patients may result from multiple interrelated factors. Intervention strategies may exert beneficial effects by improving vascular function, reducing inflammation, or enhancing neuroprotective pathways [46,47].

Our predictive modeling showed that diabetes consistently predicted enhanced response, while hypertension showed opposite effects. These findings suggest that patients with comorbidity may benefit more from intervention. The immersive cognitive-motor training may help counteract diabetes-induced neuroinflammation and vascular dysfunction through enhanced neuroplasticity, while the engaging nature of VR technology may improve treatment adherence and motivation—factors that are often compromised in patients with chronic metabolic disorders [48]. These predictive results were aligned with subgroup analysis that patients with comorbidity may benefit more from intervention modality with high confidence.

Beyond the benefits observed of individual intervention components, our study showed the importance of adaptive treatment strategies. Traditional cognitive interventions have predominantly used fixed protocols with predetermined duration and intensity, failing to account for individual variability in treatment response. The coaching-based intervention represented an early attempt at adaptive intervention [49-51], using remote support to prevent cognitive function decline. However, these adaptations remained largely experience-driven rather than systematically evidence-based. Our study addressed limitations of subjective feelings as response indicators by examining the agreement between subjective response assessment and objective assessment criteria at 12 weeks. Subjective assessment used patient-reported measures of intervention mastery and perceived effectiveness to guide treatment decisions, and objective assessment used improvement of MGs at 12 weeks. The moderate agreement (κ=0.42) between subjective response assessment and objective cognitive improvement validated our classification approach, demonstrating that participants showing both subjective benefit and objective gains could continue original protocols while nonresponders required treatment modification.

Dynamic treatment regimen analysis revealed that adaptive strategies consistently outperformed static approaches, with treatment intensification proving more effective than modality switching for nonresponders in both intervention groups. This approach represents a potential shift from an experience-based approach toward precise management, providing clinicians with evidence-based decision rules that may improve treatment efficiency and optimize resource allocation in community-based MCI prevention programs.

Further research is needed to advance this field. First, investigation of motivation and adherence factors is essential to identify predictors of intervention engagement and develop evidence-based strategies for enhancing long-term adherence to VR-based cognitive training, particularly examining how individual characteristics and intervention design features influence sustained participation and treatment response. Second, optimization of SMART design parameters is warranted, including validation of alternative response assessment time points and development of objective response criteria that integrate cognitive, physiological, and behavioral indicators to reduce reliance on subjective measures. Third, multicenter trials in diverse populations and health care settings are needed to establish the generalizability and implementation feasibility of VR-enhanced adaptive interventions, particularly examining effectiveness across different MCI subtypes and socioeconomic contexts.

### Innovations and Clinical Implications

This study advances MCI prevention research through several methodological and practical innovations. First, we developed the first SMART-based adaptive intervention framework for cognitive health, moving beyond fixed-protocol designs that dominate current practice. The 12-week objective response assessment criteria—integrating subjective mastery evaluation with cognitive performance metrics—provide clinicians with standardized decision rules for treatment modification, replacing experience-dependent judgments that vary across providers and settings. Second, our finding that treatment intensification outperforms modality switching for nonresponders establishes a specific clinical pathway: rather than abandoning ineffective interventions, clinicians should increase intervention dose before switching modalities. Third, the identification of diabetes as a positive predictor and hypertension as a negative predictor of treatment response enables risk stratification and personalized intervention selection prior to treatment initiation.

The clinical utility of these findings extends beyond research settings. Community health workers can implement the standardized decision rules without specialized neuropsychological training, as the response assessment requires only basic cognitive screening tools already deployed in Chinese primary care. The VR-Taichi protocol leverages culturally familiar mind-body exercises, addressing adherence barriers that limit Western-developed interventions in Asian populations. Our subgroup analyses identify priority populations for targeted screening: adults aged 60-71 years, individuals with MoCA scores indicating lower baseline function, and patients with metabolic comorbidities. These groups demonstrated 15%-25% greater cognitive improvement compared with their counterparts, suggesting that resource-limited settings should prioritize these populations for intensive intervention.

This precision medicine approach addresses a critical gap in current dementia prevention guidelines, which provide broad recommendations without specifying how to tailor interventions to individual characteristics or when to modify treatment based on early response. Our findings support policy development for technology-integrated cognitive health programs within urban community health systems, with potential adaptation to other metropolitan areas possessing similar digital infrastructure and hierarchical medical delivery systems.

### Implementation Feasibility and Public Health Applications

Our findings support the feasibility of implementing standardized VR-based protocols within existing community health service centers, leveraging Shanghai’s robust digital infrastructure and established hierarchical medical system. The demonstrated effectiveness of adaptive treatment strategies, particularly the superior performance of VRTC-based interventions across diverse participant characteristics, suggests that technology-enhanced approaches can be successfully integrated into routine community-based MCI prevention programs. The identification of diabetes as a positive predictor and hypertension as a negative predictor of treatment response provides practical guidance for community health workers in optimizing intervention selection and resource allocation.

The scalability of our intervention model is supported by several practical considerations specific to urban community settings. First, the Taichi protocol used in VRTC is culturally familiar to Chinese older populations, facilitating acceptance and adherence. Second, the objective response assessment criteria developed through our SMART design provide clear decision rules that can be implemented by trained community health personnel without requiring specialized clinical expertise. Third, the significant effect sizes observed suggest that even with some implementation variability expected in real-world settings, meaningful cognitive benefits are likely to be maintained. These findings support the development of evidence-based public service recommendations for integrating VR technology into Shanghai’s community health infrastructure, with potential for adaptation to other metropolitan areas with similar health care delivery systems and technological capabilities.

### Limitations

Our study has several limitations that should be considered. The sample was predominantly recruited from urban communities in Shanghai, which may limit generalizability to rural populations or different health care settings. Additionally, while our follow-up period was adequate for demonstrating intervention effects, longer-term outcomes remain to be evaluated. Subjective evaluation of intervention content mastery as a criterion for assessing participants’ responsiveness is not comprehensive, especially for aerobic exercises such as Taichi. Although robustness analysis showed no significant difference between the 2 response definitions, we still recommended that future evaluations incorporate physiological, cognitive, psychological, and behavioral indicators to fully capture participants’ responses to the intervention. Assessment based on VO₂ max, Geriatric Depression Scale (GDS), and quality-of-life measures is suggested as a robust approach for evaluating the effectiveness of nonsubjective interventions.

### Conclusion

This study represents the SMART design to develop evidence-based adaptive intervention strategies for MCI prevention in community settings, demonstrating that technology-enhanced dual-task training with VR significantly outperforms traditional exercise modalities and control conditions. The CT+VRTC intervention achieved substantial cognitive improvements (Cohen *d*=1.425, 95% CI 0.785-2.060; *P*<.001) that exceeded those reported in previous multidomain trials, with adaptive treatment strategies consistently outperforming static approaches across diverse participant characteristics. Our predictive modeling revealed that individuals with diabetes showed enhanced treatment responsiveness across all outcome measures, while those with hypertension demonstrated reduced response, highlighting the critical importance of comorbidity-informed treatment selection. The systematic approach to treatment adaptation provides clinicians with evidence-based decision rules for optimizing intervention delivery in real-world settings. These findings establish a foundation for precision-based cognitive health management, potentially bridging the gap between traditional multidomain approaches and personalized medicine strategies while offering a scalable, cost-effective solution for community-based MCI prevention programs.

These findings establish a foundation for precision-based cognitive health management, potentially bridging the gap between traditional multidomain approaches and personalized medicine strategies while offering a scalable, cost-effective solution for community-based MCI prevention programs. The demonstrated large effect sizes and adaptive treatment protocols provide evidence-based recommendations for implementing VR-enhanced cognitive interventions within Shanghai’s existing community health service infrastructure, supporting the development of technology-integrated public health strategies for urban older populations.

## Appendix

Multimedia Appendix 1 CONSORT checklist.

Multimedia Appendix 2 Detailed Intervention Protocol.

Multimedia Appendix 3 VRTC Video.

## Abbreviations

CONSORT: Consolidated Standards of Reporting Trials
CT: cognitive training
FINGER: The Finnish Geriatric Intervention Study to Prevent Cognitive Impairment and Disability
GDS: Geriatric Depression Scale
HATICE: The Healthy Ageing Through Internet Counselling in the Elderly
MCI: mild cognitive impairment
MGs: Memory Guard score
MoCA: Montreal Cognitive Assessment
OffTC: offline Taichi
SMART: Sequential Multiple Assignment Randomized Trial
VR: virtual reality
VRTC: virtual reality Taichi

## Data Availability

The datasets generated or analyzed during this study are available from the corresponding author on reasonable request.
