# Supplementary material for: Effect of Dual-Task Training on Cognitive Function in Community-Dwelling Older Adults With Mild Cognitive Impairment: Sequential Multiple Assignment Randomized Trial
Source: J Med Internet Res. 2025 Nov 25;27:e79274. doi: 10.2196/79274 (PMC12690282; doi:10.2196/79274)
Supplement: Multimedia Appendix 2 [file jmir_v27i1e79274_app2.docx]

Effect of Dual-Task Training on Cognitive Function in Community-Dwelling Older Adults With Mild Cognitive Impairment: A Sequential Multiple Assignment Randomized Trial

Chenhao Yu^1,4 +^, Dongsheng Bian^2, +^, Jiatao Zhang^1,4^, Xiao Han^1,4^, Chenshu Shi^3, *^, Guohong Li^1,3,4 *^

^1^ School of Public Health, Shanghai Jiao Tong University School of Medicine, Shanghai, 200025, China.

^2^ Department of Clinical Nutrition, Ruijin Hospital, Shanghai Jiao Tong University School of Medicine, Shanghai, 200025, China.

^3^ Institute of Healthy Yangtze River Delta, Shanghai Jiao Tong University, Shanghai, 200240, China

^4^ School of Global Health, Shanghai Jiao Tong University School of Medicine, Shanghai, 200025, China

^+^ Share the same contribution

^*^ Corresponding email address:

shihchenshu@hotmail.com

guohongli@sjtu.edu.cn

# Trail Protocol

## Principal Investigator/Faculty Advisor

Name: Guohong Li

Department: School of Public Health, Shanghai Jiao Tong University School of Medicine

Email Address: guohongli@sjtu.edu.cn

## Abbreviations

1. SMART/Sequential, multiple assignment, randomized trial
2. cDTR/Causality-driven dynamic treatment regime
3. MCI/Mild cognitive impairment
4. CT/Cognitive training
5. VRTC/Virtual reality Taichi
6. OffTC/Offline Taichi
7. SHAP/Shapley Additive explanations
8. SAC/Soft Actor-Critic
9. PI/Principal investigator

## Objectives

Mild cognitive impairment (MCI) represents a critical window for dementia prevention, yet current clinical guidelines lack specific guidance for individualized interventions despite substantial heterogeneity in treatment responses. This study uses a sequential, multiple assignment, randomized trial (SMART) design to identify optimal adaptive intervention strategies for older individuals with MCI using a causality-driven dynamic treatment regime framework (cDTR-SMART).

The chronic and relapsing nature of cognitive decline makes MCI uniquely suited to this methodology. It incorporates stepping therapy up for those who do not fully respond to initial intervention with intensified interventions, a tactic successfully applied to a number of complex chronic problems but not formally tested for MCI. The study will also examine how individual characteristics influence treatment effectiveness to enable personalized intervention strategies.

## Background

The increased life expectancy is one of the greatest achievements of global health systems. However, this increase has led to a substantial rise in age-related neurological disorders, particularly Alzheimer's disease and other dementias, necessitating health policies to focus[1], not only on survival but also on minimising health loss due to disability by promoting function and independence[2]. By 2021, dementia ranked as one of the top ten neurological conditions globally, with particularly high burden in people aged 80 years and older, second only to stroke. Between 1990 and 2021, the disability-adjusted life-years (DALYs) attributed to dementia increased by 168·7% (95% UI 156·3-179·9), driven by population ageing[3]. MCI represents a clinically subtle yet critical transitional state between normal ageing and dementia.[4] Despite its insidious onset and often unrecognized early symptoms, MCI carries significant prognostic implications, with 10-15% of affected individuals progressing to dementia annually—a risk 3·3 times higher than that of the general elderly population[5]. Therefore, MCI represents a critical window for dementia prevention and intervention, with early identification and intervention being essential for reducing dementia incidence.

In China, which has the world's largest ageing population with 280 million people aged 60 years or older (19·8% of total population) at the end of 2022, this demographic transition presents unprecedented challenges for dementia care delivery[6]. The prevalence of MCI among adults aged 60 years and older is 15·5%, affecting approximately 38·77 million individuals (95% CI 37·95-39·62), with higher rates observed in women and rural areas[7]. Research suggests that delaying dementia onset or progression by one year could reduce dementia cases by 9·2 million by 2050[7].

Evidence suggests that the optimal sequence and dose of interventions vary among individuals, with substantial heterogeneity in treatment responses[8]. Current clinical guidelines and expert consensus across different countries provide only broad, population-level recommendations for MCI and dementia prevention, lacking specific guidance for individualization in community and clinical practice[1,9–11]. Although clinicians typically adjust therapeutic strategies based on patients' conditions and treatment responses, the subtle onset and progression of MCI poses challenges for precise intervention matching[12]. Suboptimal intervention timing or inappropriate strategy selection might reduce treatment adherence and compromise the critical intervention window[13]. To address these challenges, we proposed an optimal adaptive intervention framework based on causal dynamic treatment regimes. Using a sequential, multiple assignment, randomised trial design, we identified an optimal adaptive intervention strategy specifically tailored for the community elderly population.

SMART designs often involve restricted participant populations under controlled conditions, making it challenging to extrapolate optimal intervention intensities to diverse real-world settings. This external validity concern is particularly relevant for MCI interventions, where population heterogeneity and varying environmental factors significantly influence treatment responses. To address these limitations, causal discovery methods offer potential improvements in model generalizability by explicitly accounting for underlying causal mechanisms that drive intervention effectiveness across different populations. Furthermore, conventional Dynamic Treatment Regimes (DTR) using Q-learning algorithms face methodological challenges in optimizing dual-task interventions common in cognitive rehabilitation. The approach struggles to capture non-linear interactions between different intervention intensities, potentially missing optimal combinations that could maximize therapeutic benefit. The complexity of cognitive and behavioural interventions for MCI necessitates more sophisticated approaches that can handle such non-linear relationships while maintaining interpretability for clinical implementation.

This methodological gap prompted our development of a causality-driven deep reinforcement learning framework that can effectively compute personalized intervention intensities for dual-task interventions while accounting for individual patient characteristics and temporal dynamics.

### Evidence before this study

Dementia is one of the leading causes of dependency and disability among older adults worldwide, affecting more than 57 million people with associated global healthcare costs[14]. The number of people with dementia is projected to increase nearly threefold to 153 million by 2050, with particularly concerning impact in China due to rapid population aging. Mild cognitive impairment (MCI) is a transitional state between normal aging and dementia, affecting approximately 38.77 million Chinese adults aged 60 years and older, with 10-15% progressing to dementia annually[11,15]. Early intervention in MCI stage is critical for delaying progression to dementia. Key interventions include cognitive stimulation, physical activity, vascular risk management, and addressing modifiable lifestyle factors such as diet, smoking cessation, and social engagement[1,16,17]. Despite the well-documented physiological and health benefits of physical exercise, the evidence supporting its impact on cognitive function remains controversial. We searched PubMed, Web of Science, and PsychInfo databases for randomized controlled trials published between database inception and April 1, 2024, focusing on long-term (≥12 months) physical exercise or multidomain interventions in non-demented, community-dwelling older adults. Previous meta-analyses examining exercise interventions have shown inconsistent results, with some reporting moderate positive effects on cognitive function in older adults, while others found negligible or no effects after accounting for publication bias and methodological limitations. Recent systematic reviews suggest that sustained multidomain interventions incorporating physical exercise may be more beneficial than exercise alone in preventing cognitive decline[18–26], particularly in individuals at high risk of dementia. Recent evidence indicates that interventions demonstrate a clear dosage-effect relationship, with optimal benefits occurring at specific thresholds that vary by individual characteristics such as age and cognitive status[27]. However, current clinical guidelines and expert consensuses across countries provide only broad intervention recommendations, lacking specificity and practical operability for clinical implementation[11]. The optimal type and duration of exercise for potentially preventing cognitive decline or dementia onset remains unclear, with considerable variation in exercise protocols observed across studies. Therefore, developing analytical frameworks to determine optimal intervention strategies and their implementation represents a critical gap in current research. Additionally, the comparative effectiveness of single-domain exercise interventions versus comprehensive multidomain approaches in preserving cognitive function and reducing dementia risk has not been systematically evaluated in long-term trials. More importantly, research specifically examining optimal adaptive intervention frameworks on MCI remains none, with most studies using fixed intervention protocols rather than dynamic, personalized approaches.

### Added value of this study

This study represents the first exploration of adaptive optimal intervention strategies in MCI management through a causality-driven dynamic treatment regime framework by sequential, multiple assignment, randomized trial (cDTR-SMART). The cDTR-SMART is a generalizable computational framework for deriving adaptive optimal intervention strategies, which integrates Peter-Clark causal discovery algorithms to identify influential causal pathways and incorporates them into a state space model, ultimately utilizing reinforcement learning to determine optimal action spaces across different states.

In our analysis of participants, we identified cognitive status, age, and presence of diabetes and hypertension as key state space variables, which enabled the derivation of personalized optimal intervention combinations (Virtual reality Taichi, Offline Taichi, Cognitive function training) through our reinforcement learning model. The study revealed substantial individual heterogeneity in treatment responses, with an average treatment effect of 0·587 (95% CI [0·022, 1·152]). Notably, the intervention demonstrated pronounced effectiveness in older participants with lower cognitive states (ITE: 0.94), with this effect further amplified in the presence of chronic conditions (ITE: 1.12), suggesting that the most vulnerable subgroups might benefit most from the intervention. Additionally, SHAP analysis revealed that age demonstrated the most pronounced heterogeneous effects in VR Taichi responses, while diabetes status showed consistently positive clustering of SHAP values for offline Taichi practice, indicating enhanced benefits for participants with metabolic conditions. This data-driven approach transcends the generic non-pharmacological intervention guidelines in current dementia prevention protocols by providing actionable, precise intervention recommendations. Furthermore, by incorporating causal discovery methods, this approach offers interpretable insights into intervention intensity adjustments based on individual patient characteristics, making it both practically applicable and theoretically sound for personalized cognitive intervention.

### Implications of all the available evidence

The mounting prevalence of dementia presents an urgent public health challenge that demands immediate attention. While current clinical guidelines acknowledge the importance of early intervention, they often lack specific, actionable recommendations for personalized treatment. Our study bridges this critical gap by providing evidence-based, optimal adaptive strategies through the innovative cDTR-SMART framework. Notably, our findings highlight the crucial role of comorbid chronic diseases in MCI intervention outcomes, demonstrating that incorporating these factors can significantly enhance treatment effectiveness. The decision tree model derived from our analysis offers practical, implementable guidance for community-based MCI prevention and management. Furthermore, the compatibility of our intervention framework with existing mobile health platforms enables real-time, precision-based adaptive interventions, making it particularly valuable for widespread community implementation. Beyond MCI, the cDTR-SMART framework represents a versatile methodology that could be adapted for developing personalized interventions across various chronic conditions, potentially transforming the approach to precision medicine in community healthcare settings.

## SMART Design

A Sequential Multiple Assignment Randomized Trial (SMART) design is a multistage trial methodology specifically developed to inform the construction of effective adaptive interventions[28–31]. Adaptive interventions are sequences of decision rules that recommend when and how to modify interventions based on individuals' characteristics and evolving response to treatment. Unlike traditional randomized controlled trials (RCTs) that evaluate fixed interventions, SMART designs enable researchers to systematically test critical questions in the development of adaptive interventions, particularly questions about treatment sequencing and individualization[29].

The SMART design is particularly appropriate for our research objectives for several reasons:

### Addressing Heterogeneity in Treatment Response

Mild cognitive impairment (MCI) patients demonstrate significant heterogeneity in disease progression, cognitive profiles, and responses to interventions[32]. Current evidence suggests that the optimal sequence and dosage of interventions vary substantially among individuals, with important implications for intervention effectiveness. The SMART design explicitly addresses this heterogeneity by enabling evaluation of multiple intervention pathways tailored to individual response patterns.

For example, as shown in our preliminary data, participants with different baseline cognitive statuses and comorbidity profiles respond differently to VR-based Taichi versus conventional Taichi. A SMART design allows us to systematically investigate these differential response patterns and develop evidence-based rules for matching patients to the most beneficial intervention sequences.

### Enabling Dynamic Decision-Making

MCI management requires making sequential treatment decisions over time, particularly as patients' cognitive status changes[33,34]. The SMART design mirrors this clinical reality by incorporating decision points where intervention strategies can be modified based on interim outcomes. This approach allows us to answer critical questions about when to intensify, switch, or maintain interventions based on early response indicators.

Specifically, our design includes an assessment of intervention mastery at week 12, with non-responders being re-randomized to either alternative interventions (switching from offline Taichi to VR Taichi or vice versa) or intensified interventions (doubled doses). This mirrors clinical practice where practitioners typically adjust treatment approaches when patients show insufficient progress.

### Identifying Optimal Treatment Sequencing

The optimal ordering of multicomponent interventions is a critical but understudied aspect of MCI management. A SMART design can determine whether treatment sequences matter - for instance, whether initiating with VR Taichi followed by offline Taichi for non-responders produces different outcomes compared to the reverse sequence.

This is particularly relevant given recent evidence suggesting potential synergistic effects between different intervention modalities. For example, Boa Sorte Silva et al. (2024) and has demonstrated that combined exercise and cognitive interventions may yield greater benefits than single-component interventions[35].

### Overcoming Limitations of Traditional Methodology

Conventional approaches to studying adaptive interventions have significant limitations that SMART designs overcome:

1. **Observational studies** that inform current adaptive intervention practices lack the randomization necessary for causal inference, making their findings vulnerable to confounding.
2. **Multiple single-stage RCTs** conducted in sequence would require substantially more participants, time, and resources than an integrated SMART. Moreover, they would fail to capture the important interaction effects between sequential treatments that may influence long-term outcomes.
3. **Standard factorial designs** are inefficient for studying adaptive interventions as they do not account for the time-varying nature of treatment decisions and cannot easily incorporate response-contingent treatment adaptations.

### Integration with Causal Discovery Methods

Our study innovatively integrates SMART with causal discovery methods to develop a causality-driven dynamic treatment regime framework (cDTR-SMART). This methodological approach enhances model generalizability by explicitly accounting for underlying causal mechanisms while effectively capturing complex non-linear relationships between intervention intensities.

This integration addresses key limitations of conventional SMART analyses:

1. It reduces the effective dimensionality of the problem, enabling valid inference with smaller sample sizes - particularly important for MCI research where large-scale recruitment can be challenging[36].
2. It captures complex, non-linear dose-response relationships in multicomponent interventions that linear models might miss.

## Study Endpoints/Events/Outcomes

## Primary Aim

Determine the optimal adaptive intervention strategy for older individuals with MCI using a causality-driven dynamic treatment regime framework.

## Secondary Aim

Evaluate the average treatment effects (ATE) of different intervention modalities (virtual reality Taichi, offline Taichi, and cognitive training) on cognitive scores measured by Memory Guard.

Analyze heterogeneity in treatment effects across participant subgroups based on cognitive status, comorbidities, and demographic characteristics.

## Primary Outcome

The primary outcome is the optimal adaptive intervention strategy derived through reinforcement learning algorithms, quantifying personalized temporal allocations for virtual reality Taichi (VRTC), offline Taichi (OffTC), and cognitive training (CT) in clinically implementable 5-minute intervals.

## Secondary Outcomes

Average treatment effects (ATE) of cognitive scores measured by Memory Guard.

Individual treatment effects (ITEs) across participant subgroups with different baseline characteristics.

## Sample Size and Power Analysis

The study size was determined based on response rate parameters drawing from multicomponent intervention studies and previous response rates. We calculated a required sample of 54 participants in the intervention group (assuming 0.60 response rate, 0.05 type I error, 0.45 half-width confidence interval, and 0.80 statistical power).

## Study Intervention

This study employs a sequential, multiple assignment, randomized trial (SMART) design to compare adaptive interventions for MCI. Participants will be randomly assigned to either control (receiving health education and routine care) or intervention groups receiving cognitive training with offline Taichi or virtual reality Taichi.

Non-responders at 12 weeks (defined as those unable to master the intervention content) will be re-randomized to alternative or intensified interventions. The study will incorporate stepping therapy up for those who continue to show cognitive decline with intensified interventions.

### Intervention Components

OffTC intervention will implement the standardized 24-form simplified Taichi exercise regimen, developed by the National Sports Administration in 1956 based on Yang-style Taichi principles. Modifications will be implemented for balance-intensive movements to enhance safety while maintaining form integrity.

VRTC intervention will utilize identical 24-form simplified Taichi movements implemented through an immersive technological interface. The VR system will incorporate precise arm-length calibration to accurately track upper and lower extremity movement trajectories.

Application: Guided Tai Chi (Developer: Cubicle Ninjas, Version 1.2.74, Release date: May 21, 2019)

Platform: Meta Quest VR Headsets.

[Video Supplementary files]


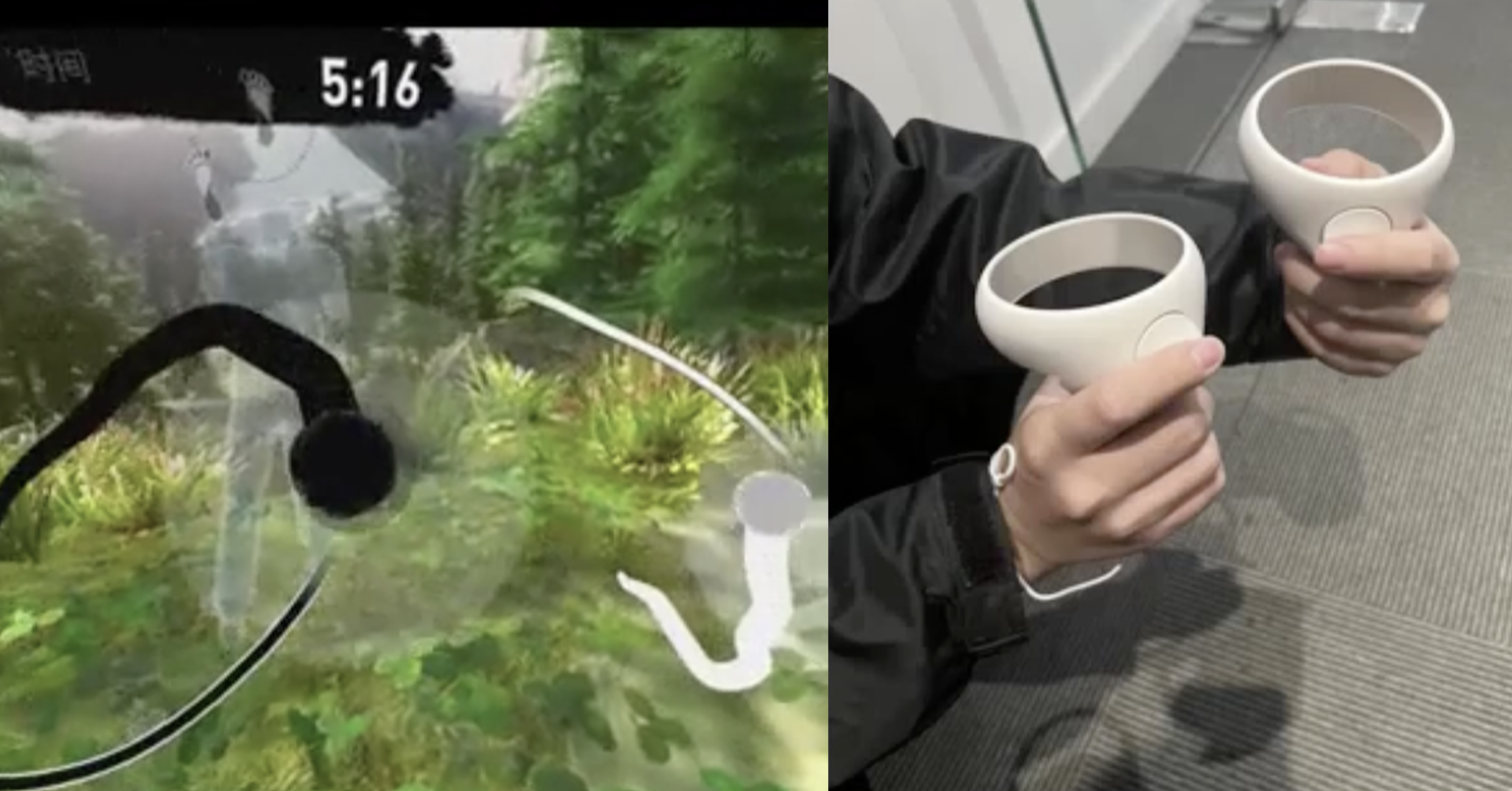


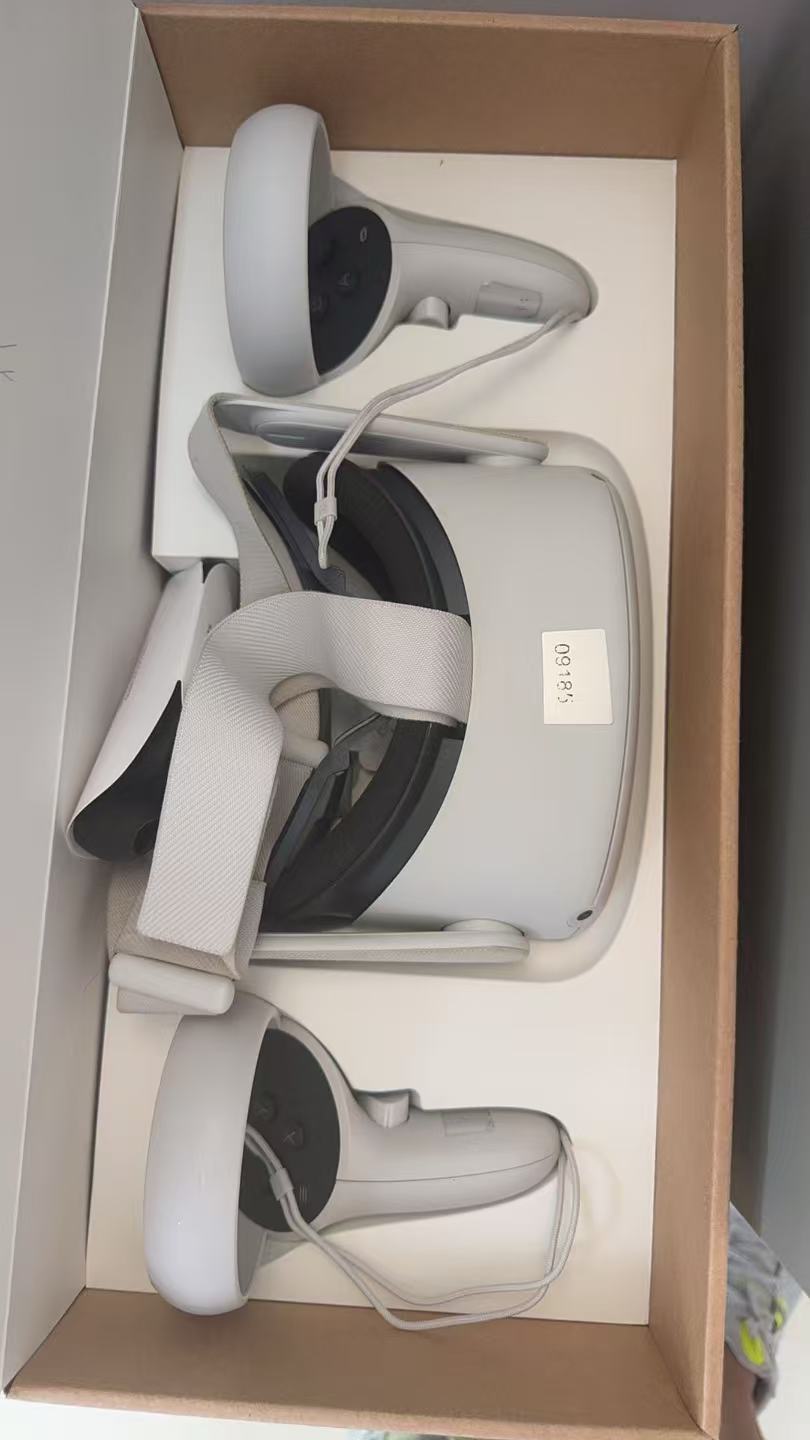
CT will utilize a comprehensive platform targeting multiple cognitive domains: memory, attention, executive function, logical reasoning, and reaction time. The system will be implemented via a customized WeChat mini-program interface.


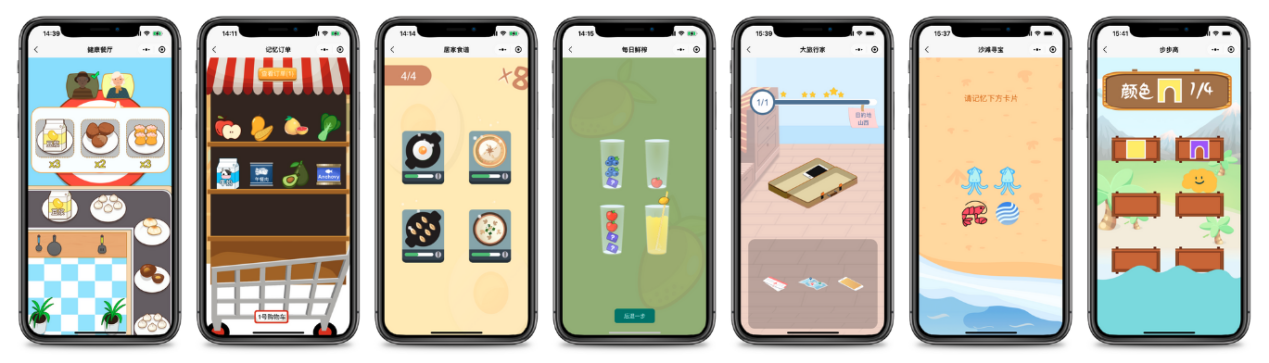


## Procedures Involved

This study employs a prospective sequential, multiple assignment, randomized trial design. The 24-week SMART design comprises two 12-week phases. In the first phase, eligible participants will be randomly assigned to receive either offline Taichi plus cognitive training (OffTC+CT) or virtual reality Taichi plus cognitive training (VRTC+CT). Each intervention will consist of 1-hour weekly exercise combined with 1 hour weekly cognitive training.

At week 12, participants will be assessed for intervention mastery, with non-responders (defined as those subjectively unable to master the intervention content, scoring less than 3 on a 5-point Likert scale) being re-randomized to alternative or intensified interventions for the second 12-week phase. Responders in Group OffTC+CT will continue their original intervention protocol, while non-responders will be randomly assigned to either VRTC+CT or doubled doses of their original intervention (2OffTC+2CT). Similarly, responders in Group VRTC+CT will maintain their initial protocol, while non-responders will be randomly assigned to either OffTC+CT or doubled doses of their original intervention (2VRTC+2CT). The control group will receive only health education and routine care throughout the 24-week period.

The anticipated duration for individual participation in the study is 24 weeks (6 months). The duration anticipated to enroll all study participants is approximately 9 months (April to December 2023).


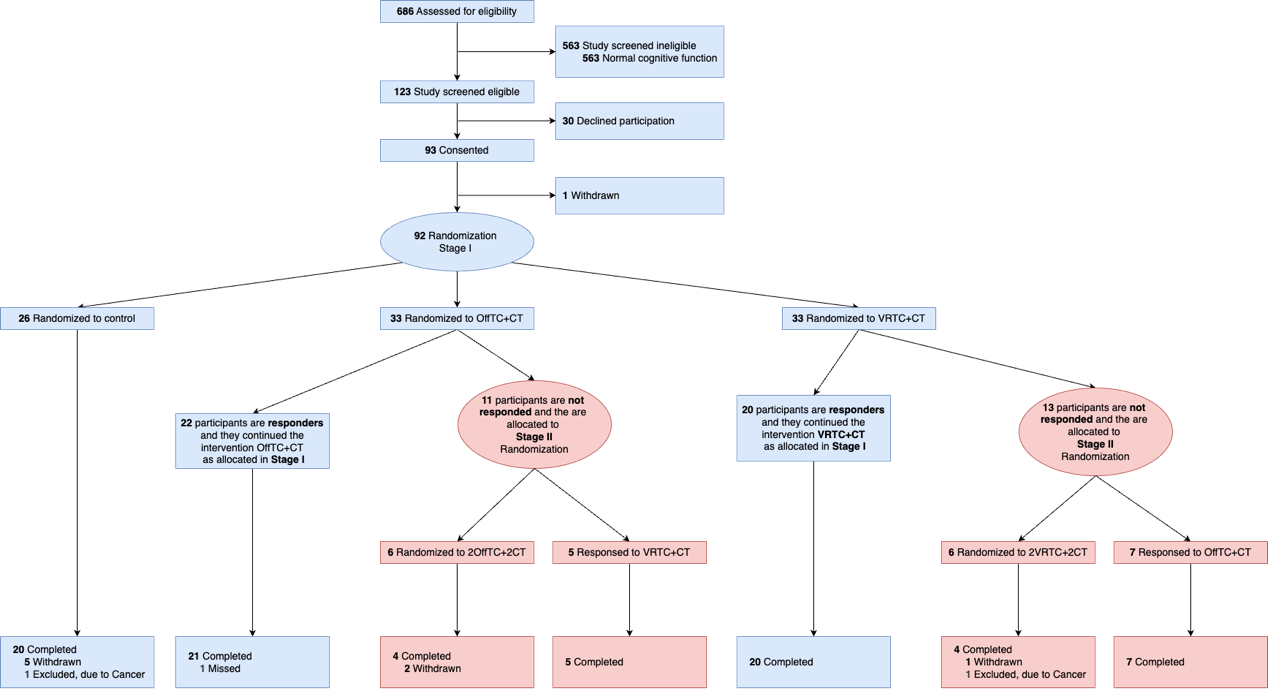


### Study Procedures

Registration to the study will occur after the subject consent is signed and eligibility is confirmed. Participants will be assigned to treatment groups using computer-generated randomization schemes, stratified by site. The first randomization will occur at baseline after the consent process is complete.

**First-Phase Intervention (Weeks 1-12)**

All participants assigned to intervention groups will receive either:

Offline Taichi plus cognitive training (OffTC+CT): 1-hour weekly OffTC sessions plus 1-hour weekly cognitive training

Virtual reality Taichi plus cognitive training (VRTC+CT): 1-hour weekly VRTC sessions plus 1-hour weekly cognitive training

The control group will receive health education and routine care.

**Assessment of Response (Week 12)**

At week 12, participants will be assessed for intervention mastery using a 5-point Likert scale. Those scoring less than 3 will be considered non-responders.

**Second-Phase Intervention (Weeks 13-24)**

Responders will continue their original intervention protocol. Non-responders will be re-randomized to either:

Alternative intervention: Switching from OffTC+CT to VRTC+CT or vice versa

Intensified intervention: Doubled doses of their original intervention (2OffTC+2CT or 2VRTC+2CT)

### Outcome Assessment

Participants' cognitive function will be monitored using the Memory Guard assessment tool at predetermined intervals (baseline, week 12, and week 24).


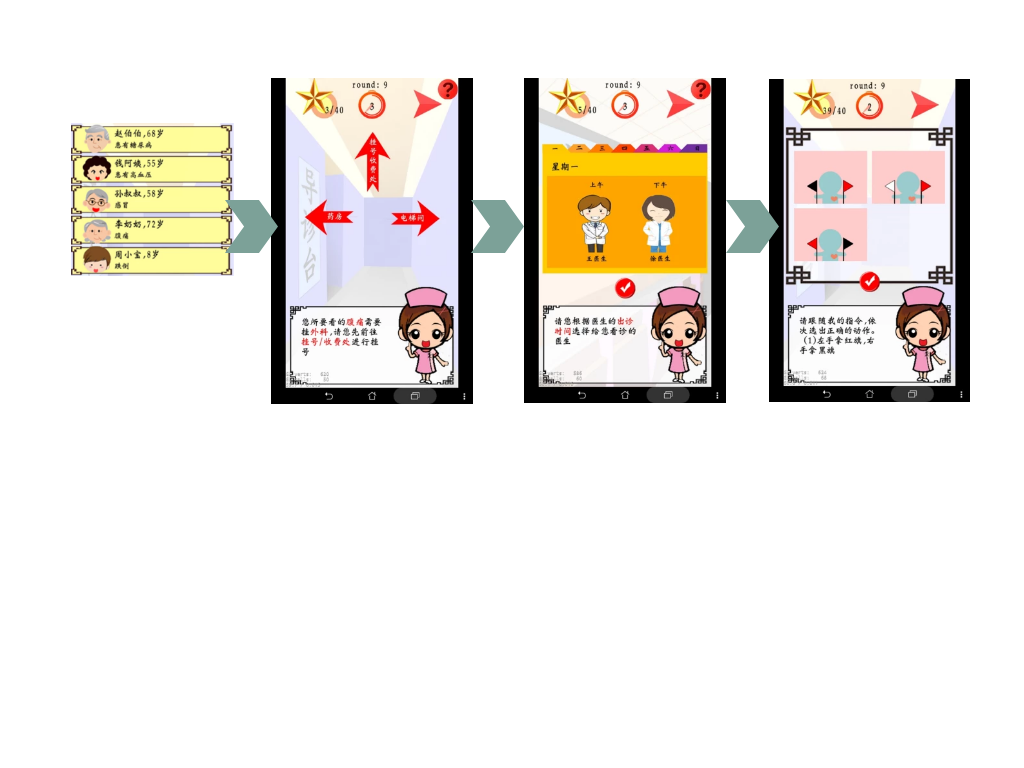


## Data and Specimen Banking

No specimens will be collected or banked for this study.

## Sharing of Results with Participants

This study does not involve diagnostic or genetic tests which could lead to the discovery of incidental findings that would need to be shared with the participant and/or the participant's medical team. The results of the cognitive assessments will be shared with participants at the conclusion of the study if requested.

## Study Population

### Inclusion Criteria

1. Age ≥60 years
2. MCI diagnosis established through positive screening on the Memory Guard assessment tool and confirmed by MoCA score <26
3. Demonstrated ability to complete assessments independently
4. Residence in the local community
5. Proficiency in smartphone operation

### Exclusion Criteria

1. Communication disorders
2. Severe impairment in activities of daily living
3. Presence of metal implants (e.g., cardiac pacemakers, artificial heart valves)
4. Severe psychiatric disorders
5. Illiteracy
6. Cognitive decline attributable to other pathologies (cerebrovascular disease, CNS infection, neurodegenerative disorders, traumatic brain injury, intracranial lesions, endocrinopathies, or vitamin deficiencies)
7. History of neurological diseases
8. Psychiatric disorders meeting DSM-5 criteria
9. Exercise contraindications
10. Color blindness
11. Insufficient education to complete testing protocols
12. Concurrent rehabilitation therapy
13. Regular exercise experience (e.g., Taichi, Baduanjin) within the preceding 3 months

### Screening

Potential participants will be screened using the Memory Guard assessment tool and the Montreal Cognitive Assessment (MoCA). Those with a positive screening on Memory Guard will be considered for inclusion. MoCA score <26 will be conducted to confirm eligibility based on the inclusion and exclusion criteria listed above.

## Vulnerable Populations

## Local Number of Participants

92 participants will be enrolled (approximately 30 each for OffTC+CT and VRTC+CT, and approximately 30 for control).

## Local Recruitment Methods

Community-dwelling older adults from three randomly selected districts in Shanghai will be systematically screened for MCI. Eligible individuals will be approached and provided with detailed information about the study.

Potential participants will be identified through community health centers, senior centers, and other venues serving older adults. Screening for MCI will be conducted using the Memory Guard assessment tool followed by the Montreal Cognitive Assessment (MoCA).

Participants will not receive financial compensation for participation, but all interventions will be provided at no cost.

## Withdrawal of Participants

Participants may withdraw from the study at any time without penalty. Investigators may withdraw participants if they develop conditions that meet exclusion criteria or if continued participation poses a risk to the participant's health or well-being.

Participants who elect to withdraw from the study will be asked to provide a reason for withdrawal, which will be documented. If possible, a final assessment will be conducted at the time of withdrawal.

## Risks to Participants

The potential risks associated with this study are minimal. Taichi exercise may cause mild muscle soreness or fatigue, particularly in participants who are not accustomed to regular physical activity. Virtual reality Taichi may cause mild dizziness or disorientation in some participants. These symptoms are typically temporary and resolve with rest.

The cognitive training component poses minimal risk but may cause mild frustration or fatigue in some participants. There is also a small risk of loss of confidentiality.

## Potential Benefits to Participants

Participants may benefit from improvements in cognitive function and physical health as a result of the interventions. Even if participants do not directly benefit, the knowledge gained from this study may help improve treatments for MCI in the future.

## Statistical Considerations

**Average Treatment Effects**

We will use causal forests double machine learning to estimate:

Total ATE between treatment and control groups on cognitive outcomes

Component-specific ATE for VRTC, OffTC, and CT

**Heterogeneity in Treatment Effects**

We will analyze heterogeneity in treatment effects through:

SHAP value analysis to quantify the impact of participant characteristics on treatment effectiveness

## Confidentiality

Data for this study will be entered into a secure database with data checks used during data entry to ensure data quality. Access to the study's data will be restricted to members of the study team by username and password. Research data will be collected from the participant and entered into the database only after written informed consent is obtained.

## Provisions to Monitor the Data to Ensure the Safety of Participants

The principal investigator and study team will assume responsibility for monitoring enrollment, adverse events, data completeness, outcome data, protocol non-compliance, and new and relevant information.

## Provisions to Protect the Privacy Interests of Participants

All research staff will receive training on protecting participant privacy and confidentiality. Interviews and assessments will be conducted in private settings. Data will be de-identified to the extent possible for analysis.

## Consent Process

All potential study participants will be given detailed information about the study in both written and verbal formats. The investigator or designee will explain all aspects of the study in lay language and answer all questions. If the participant decides to participate, they will be asked to sign and date the consent document.

## Setting

The study will be conducted in three randomly selected districts in Shanghai, China. Intervention sessions will take place at community health centers or other suitable venues with appropriate facilities for the Taichi interventions and cognitive training.

## References

1. Livingston G, Huntley J, Liu KY, Costafreda SG, Selbæk G, Alladi S, Ames D, Banerjee S, Burns A, Brayne C, Fox NC, Ferri CP, Gitlin LN, Howard R, Kales HC, Kivimäki M, Larson EB, Nakasujja N, Rockwood K, Samus Q, Shirai K, Singh-Manoux A, Schneider LS, Walsh S, Yao Y, Sommerlad A, Mukadam N. Dementia prevention, intervention, and care: 2024 report of the Lancet standing Commission. The Lancet Elsevier; 2024 Aug 10;404(10452):572–628. PMID:39096926

2. Chen X, Giles J, Yao Y, Yip W, Meng Q, Berkman L, Chen H, Chen X, Feng J, Feng Z, Glinskaya E, Gong J, Hu P, Kan H, Lei X, Liu X, Steptoe A, Wang G, Wang H, Wang H, Wang X, Wang Y, Yang L, Zhang L, Zhang Q, Wu J, Wu Z, Strauss J, Smith J, Zhao Y. The path to healthy ageing in China: a Peking University–Lancet Commission. The Lancet Elsevier; 2022 Dec 3;400(10367):1967–2006. PMID:36423650

3. Nandi A, Counts N, Chen S, Seligman B, Tortorice D, Vigo D, Bloom DE. Global and regional projections of the economic burden of Alzheimer’s disease and related dementias from 2019 to 2050: A value of statistical life approach. eClinicalMedicine Elsevier; 2022 Sept 1;51. PMID:35898316

4. Frizzell TO, Glashutter M, Liu CC, Zeng A, Pan D, Hajra SG, D’Arcy RCN, Song X. Artificial intelligence in brain MRI analysis of Alzheimer’s disease over the past 12 years: A systematic review. Ageing Research Reviews 2022 May 1;77:101614. doi: 10.1016/j.arr.2022.101614

5. Petersen RC, Lopez O, Armstrong MJ, Getchius TS, Ganguli M, Gloss D, Gronseth GS, Marson D, Pringsheim T, Day GS, Sager M, Stevens J, Rae-Grant A. Practice guideline update summary: Mild cognitive impairment: Report of the Guideline Development, Dissemination, and Implementation Subcommittee of the American Academy of Neurology. Neurology 2018 Jan 16;90(3):126. PMID:29282327

6. Jia L, Du Y, Chu L, Zhang Z, Li F, Lyu D, Li Y, Zhu M, Jiao H, Song Y, Shi Y, Zhang H, Gong M, Wei C, Tang Y, Fang B, Guo D, Wang F, Zhou A, Chu C, Zuo X, Yu Y, Yuan Q, Wang W, Li F, Shi S, Yang H, Zhou C, Liao Z, Lv Y, Li Y, Kan M, Zhao H, Wang S, Yang S, Li H, Liu Z, Wang Q, Qin W, Jia J, Quan M, Wang Y, Li W, Cao S, Xu L, Han Y, Liang J, Qiao Y, Qin Q, Qiu Q. Prevalence, risk factors, and management of dementia and mild cognitive impairment in adults aged 60 years or older in China: a cross-sectional study. The Lancet Public Health Elsevier; 2020 Dec 1;5(12):e661–e671. PMID:33271079

7. Brookmeyer R, Johnson E, Ziegler-Graham K, Arrighi HM. Forecasting the global burden of Alzheimer’s disease. Alzheimer’s & dementia Elsevier; 2007;3(3):186–191.

8. Eccles MP, Mittman BS. Welcome to Implementation Science. Implementation Science 2006 Feb 22;1(1):1. doi: 10.1186/1748-5908-1-1

9. Dunne RA, Aarsland D, O’Brien JT, Ballard C, Banerjee S, Fox NC, Isaacs JD, Underwood BR, Perry RJ, Chan D, Dening T, Thomas AJ, Schryer J, Jones A-M, Evans AR, Alessi C, Coulthard EJ, Pickett J, Elton P, Jones RW, Mitchell S, Hooper N, Kalafatis C, Rasmussen JGC, Martin H, Schott JM, Burns A. Mild Cognitive Impairment: the Manchester consensus. Age and Ageing 2020 Nov 17;50(1):72. PMID:33197937

10. Petersen RC, Lopez O, Armstrong MJ, Getchius TS, Ganguli M, Gloss D, Gronseth GS, Marson D, Pringsheim T, Day GS, Sager M, Stevens J, Rae-Grant A. Practice guideline update summary: Mild cognitive impairment: Report of the Guideline Development, Dissemination, and Implementation Subcommittee of the American Academy of Neurology. Neurology 2018 Jan 16;90(3):126. PMID:29282327

11. Chinese Dementia and Cognitive Impairment Diagnosis and Treatment Guidelines Writing Group, Cognitive Disorders Committee of the Neurology Branch of the Chinese Medical Doctor Association. 2018 Chinese guidelines for diagnosis and management of cognitive impairment and dementia (V): dementia therapy. Zhonghua Yi Xue Za Zhi 2018 May 8;98(17):1294–1301. doi: 10.3760/cma.j.issn.0376-2491.2018.17.003

12. Myszczynska MA, Ojamies PN, Lacoste AMB, Neil D, Saffari A, Mead R, Hautbergue GM, Holbrook JD, Ferraiuolo L. Applications of machine learning to diagnosis and treatment of neurodegenerative diseases. Nat Rev Neurol Nature Publishing Group; 2020 Aug;16(8):440–456. doi: 10.1038/s41582-020-0377-8

13. Karssemeijer EGA (Esther), Aaronson JA (Justine), Bossers WJ (Willem), Smits T (Tara), Olde Rikkert MGM (Marcel), Kessels RPC (Roy). Positive effects of combined cognitive and physical exercise training on cognitive function in older adults with mild cognitive impairment or dementia: A meta-analysis. Ageing Research Reviews 2017 Nov 1;40:75–83. doi: 10.1016/j.arr.2017.09.003

14. 2024 Alzheimer’s disease facts and figures. Alzheimer’s & dementia : the journal of the Alzheimer’s Association Alzheimers Dement; 2024 May;20(5). PMID:38689398

15. Wang Y, Liu T, Cai Y, Wu Y, Nie Y, Kuang W, Qiu P, Wan Y. The Chinese version of informant AD8 for mild cognitive impairment and dementia screening in community‐dwelling older adults. Public Health Nursing Wiley Online Library; 2023;40(2):258–265.

16. Livingston G, Huntley J, Sommerlad A, Ames D, Ballard C, Banerjee S, Brayne C, Burns A, Cohen-Mansfield J, Cooper C, Costafreda SG, Dias A, Fox N, Gitlin LN, Howard R, Kales HC, Kivimäki M, Larson EB, Ogunniyi A, Orgeta V, Ritchie K, Rockwood K, Sampson EL, Samus Q, Schneider LS, Selbæk G, Teri L, Mukadam N. Dementia prevention, intervention, and care: 2020 report of the Lancet Commission. The Lancet Elsevier; 2020 Aug 8;396(10248):413–446. PMID:32738937

17. Chen X, Giles J, Yao Y, Yip W, Meng Q, Berkman L, Chen H, Chen X, Feng J, Feng Z, Glinskaya E, Gong J, Hu P, Kan H, Lei X, Liu X, Steptoe A, Wang G, Wang H, Wang H, Wang X, Wang Y, Yang L, Zhang L, Zhang Q, Wu J, Wu Z, Strauss J, Smith J, Zhao Y. The path to healthy ageing in China: a Peking University–Lancet Commission. The Lancet 2022 Dec;400(10367):1967–2006. doi: 10.1016/S0140-6736(22)01546-X

18. Xue D, Li PW, Doris SF, Lin RS. Combined exercise and cognitive interventions for adults with mild cognitive impairment and dementia: a systematic review and network meta-analysis. International Journal of Nursing Studies Elsevier; 2023;147:104592. Available from: https://www.sciencedirect.com/science/article/pii/S0020748923001578 [accessed Mar 25, 2025]

19. Rieker JA, Reales JM, Muiños M, Ballesteros S. The effects of combined cognitive-physical interventions on cognitive functioning in healthy older adults: a systematic review and multilevel meta-analysis. Frontiers in human neuroscience Frontiers Media SA; 2022;16:838968. Available from: https://www.frontiersin.org/articles/10.3389/fnhum.2022.838968/full [accessed Mar 25, 2025]

20. Reparaz-Escudero I, Izquierdo M, Bischoff-Ferrari HA, Martínez-Lage P, de Asteasu MLS. Effect of long-term physical exercise and multidomain interventions on cognitive function and the risk of mild cognitive impairment and dementia in older adults: A systematic review with meta-analysis. Ageing Research Reviews Elsevier; 2024;102463. doi: 10.1016/j.arr.2024.102463

21. García-Llorente AM, Casimiro-Andújar AJ, Linhares DG, De Souza Vale RG, Marcos-Pardo PJ. Multidomain interventions for sarcopenia and cognitive flexibility in older adults for promoting healthy aging: a systematic review and meta-analysis of randomized controlled trials. Aging Clin Exp Res 2024 Feb 22;36(1):47. doi: 10.1007/s40520-024-02700-2

22. Furtado GE, Reis ASL da S, Braga-Pereira R, Caldo-Silva A, Teques P, Sampaio AR, Santos CAF dos, Bachi ALL, Campos F, Borges GF. Impact of Exercise Interventions on Sustained Brain Health Outcomes in Frail Older Individuals: A Comprehensive Review of Systematic Reviews. Healthcare MDPI; 2023. p. 3160. doi: 10.3390/healthcare11243160

23. Dominguez LJ, Veronese N, Vernuccio L, Catanese G, Inzerillo F, Salemi G, Barbagallo M. Nutrition, physical activity, and other lifestyle factors in the prevention of cognitive decline and dementia. Nutrients MDPI; 2021;13(11):4080. Available from: https://www.mdpi.com/2072-6643/13/11/4080 [accessed Mar 25, 2025]

24. Chen W, Siew-Pin JL, Wu Y, Huang N, Teo W-P. Identifying exercise and cognitive intervention parameters to optimize executive function in older adults with mild cognitive impairment and dementia: a systematic review and meta-analyses of randomized controlled trials. Eur Rev Aging Phys Act 2024 Aug 30;21(1):22. doi: 10.1186/s11556-024-00357-4

25. Castro CB, Costa LM, Dias CB, Chen J, Hillebrandt H, Gardener SL, Brown BM, Loo RL, Garg ML, Rainey-Smith SR. Multi-domain interventions for dementia prevention–a systematic review. The Journal of nutrition, health and aging Elsevier; 2023;27(12):1271–1280. Available from: https://www.sciencedirect.com/science/article/pii/S1279770724002240 [accessed Mar 25, 2025]

26. Ahn S, Chung JW, Crane MK, Bassett DR, Anderson JG. The Effects of Multi-Domain Interventions on Cognition: A Systematic Review. West J Nurs Res 2022 Dec;44(12):1134–1154. doi: 10.1177/01939459211032272

27. Liu L, Wang H, Xing Y, Zhang Z, Zhang Q, Dong M, Ma Z, Cai L, Wang X, Tang Y. Dose–response relationship between computerized cognitive training and cognitive improvement. npj Digit Med Nature Publishing Group; 2024 Aug 15;7(1):1–7. doi: 10.1038/s41746-024-01210-9

28. McGorry PD, Mei C, Amminger GP, Yuen HP, Kerr M, Spark J, Wallis N, Polari A, Baird S, Buccilli K, Dempsey S-JA, Ferguson N, Formica M, Krcmar M, Quinn AL, Mebrahtu Y, Ruslins A, Street R, Wannan C, Dixon L, Carter C, Loewy R, Niendam TA, Shumway M, Nelson B. A Sequential Adaptive Intervention Strategy Targeting Remission and Functional Recovery in Young People at Ultrahigh Risk of Psychosis: The Staged Treatment in Early Psychosis (STEP) Sequential Multiple Assignment Randomized Trial. JAMA Psychiatry 2023 Sept 1;80(9):875. doi: 10.1001/jamapsychiatry.2023.1947

29. Kidwell KM, Almirall D. Sequential, Multiple Assignment, Randomized Trial Designs. JAMA 2023 Jan 24;329(4):336. doi: 10.1001/jama.2022.24324

30. Wang X, Chakraborty B. The Sequential Multiple Assignment Randomized Trial for Controlling Infectious Diseases: A Review of Recent Developments. Am J Public Health 2023 Jan;113(1):49–59. doi: 10.2105/AJPH.2022.307135

31. Waring R. CD4+ Count–Guided Interruption of Antiretroviral Treatment. The new england journal of medicine 2006;

32. Ekelund U, Tarp J, Steene-Johannessen J, Hansen BH, Jefferis B, Fagerland MW, Whincup P, Diaz KM, Hooker SP, Chernofsky A, Larson MG, Spartano N, Vasan RS, Dohrn I-M, Hagströmer M, Edwardson C, Yates T, Shiroma E, Anderssen SA, Lee I-M. Dose-response associations between accelerometry measured physical activity and sedentary time and all cause mortality: systematic review and harmonised meta-analysis. BMJ 2019 Aug 21;366:l4570. PMID:31434697

33. Dunne RA, Aarsland D, O’Brien JT, Ballard C, Banerjee S, Fox NC, Isaacs JD, Underwood BR, Perry RJ, Chan D, Dening T, Thomas AJ, Schryer J, Jones A-M, Evans AR, Alessi C, Coulthard EJ, Pickett J, Elton P, Jones RW, Mitchell S, Hooper N, Kalafatis C, Rasmussen JGC, Martin H, Schott JM, Burns A. Mild Cognitive Impairment: the Manchester consensus. Age and Ageing 2021 Jan 1;50(1):72–80. doi: 10.1093/ageing/afaa228

34. Liss JL, Seleri Assunção S, Cummings J, Atri A, Geldmacher DS, Candela SF, Devanand DP, Fillit HM, Susman J, Mintzer J, Bittner T, Brunton SA, Kerwin DR, Jackson WC, Small GW, Grossberg GT, Clevenger CK, Cotter V, Stefanacci R, Wise-Brown A, Sabbagh MN. Practical recommendations for timely, accurate diagnosis of symptomatic Alzheimer’s disease (MCI and dementia) in primary care: a review and synthesis. Journal of Internal Medicine 2021;290(2):310–334. doi: 10.1111/joim.13244

35. Silva NCBS, Brinke LF ten, Bielak AAM, Handy TC, Liu-Ambrose T. Improved intraindividual variability in cognitive performance following cognitive and exercise training in older adults. Journal of the International Neuropsychological Society 2024 May;30(4):328–338. doi: 10.1017/S1355617723000577

36. Tu R, Zhang K, Kjellström H, Zhang C. Optimal transport for causal discovery. arXiv; 2022. doi: 10.48550/arXiv.2201.09366
